# The Physician’s Visiting List for 1867

**Published:** 1866-10

**Authors:** 


THE PHYSICIAN'S VISITING LIST FOR 1867. Philadelphia: LINDSAY & BLAKISTON.

This very great convenience for the practitioner has been so long and so generally in use by the profession, that its merits are well known. For sale by W. B. KEEN & Co., 148 Lake Street. Prices—For accommodating 25 patients, in cloth, 75 cents, in leather, $1.25; for 50 patients, in cloth, $1.00, in leather, $1.50.

